# A Computational Pipeline to Investigate Longitudinal Blood Flow Changes in the Circle of Willis of Patients with Stable and Growing Aneurysms

**DOI:** 10.1007/s10439-024-03493-1

**Published:** 2024-04-14

**Authors:** Alberto Coccarelli, Raoul Van Loon, Aichi Chien

**Affiliations:** 1 Zienkiewicz Institute for Modelling, Data and AI, Faculty of Science and Engineering, Swansea University, Swansea, UK; 2 Department of Mechanical Engineering, Faculty of Science and Engineering, Swansea University, Swansea, UK; 3 Biomedical Engineering Simulation and Testing Lab, Department of Biomedical Engineering, Faculty of Science and Engineering, Swansea University, Swansea, UK; 4 Radiological Sciences, School of Medicine, University of California Los Angeles (UCLA), Los Angeles, CA USA

**Keywords:** Circle of Willis, Aneurysm development, Cerebral vasculature, One-dimensional blood flow dynamics, Longitudinal study

## Abstract

Changes in cerebral blood flow are often associated with the initiation and development of different life-threatening medical conditions including aneurysm rupture and ischemic stroke. Nevertheless, it is not fully clear how haemodynamic changes in time across the Circle of Willis (CoW) are related with intracranial aneurysm (IA) growth. In this work, we introduced a novel reduced-order modelling strategy for the systematic quantification of longitudinal blood flow changes across the whole CoW in patients with stable and unstable/growing aneurysm. Magnetic Resonance Angiography (MRA) images were converted into one-dimensional (1-D) vessel networks through a semi-automated procedure, with a level of geometric reconstruction accuracy controlled by user-dependent parameters. The proposed pipeline was used to systematically analyse longitudinal haemodynamic changes in seven different clinical cases. Our preliminary simulation results indicate that growing aneurysms are not necessarily associated with significant changes in mean flow over time. A concise sensitivity analysis also shed light on which modelling aspects need to be further characterized to have reliable patient-specific predictions. This study poses the basis for investigating how time-dependent changes in the vasculature affect the haemodynamics across the whole CoW in patients with stable and growing aneurysms.

**Supplementary Information:**

The online version contains supplementary material available at 10.1007/s10439-024-03493-1.

## Introduction

Cerebral arteries in the Circle of Willis (CoW) form an interconnected vessel network for the optimal transport of nutrients and oxygen to the different brain regions. At some vascular locations, wall weakening may lead to the formation of life-threatening aneurysms, which exhibit features of inflammation and tissue degeneration [1]. Interestingly, the structural integrity of unruptured aneurysm wall is extremely variable [2] and the aetiology of these vascular lesions appears very complex [3], with aberrant levels of flow and wall shear stress playing a major role in the remodelling process [4, 5]. In clinics, brain angiograms are often used for monitoring the evolution of patient’s IA in time. The CoW is continuously affected by growth and remodelling phenomena, and therefore, follow-up scans often reveals the formation of new additional aneurysms. This poses the need for methodologies that can be promptly and easily used for a regular patient condition monitoring.

Since aneurysm onset and development is strongly influenced by the local hemodynamic conditions [6], high-fidelity/three dimensional (3-D) computational fluid dynamics models [7–10] are generally employed for studying the interplay between blood flow and vascular growth/rupture. Blood flow simulations can be used in conjunction with morphological data to identify metrics for predicting aneurysm rupture [7, 11, 12]. Since local flow changes may be compensated and/or even dictated by other neighbouring vessels, a holistic approach capable to describe vascular adaptations over time is preferable. High-fidelity models that consider the vascular wall distensible are computationally very demanding, and hence cannot be easily used for computing the haemodynamics in large portions of the CoW. Parallel blood flow solvers in combination with HPC [13] may overcome this domain-size limitation but their use in everyday clinical practice appears prohibitive.

Due to its computational efficiency, 1-D blood flow modelling could represent a valuable strategy for investigating the causative link between longitudinal flow changes and vascular adaptation across the whole CoW, and potentially identifying and monitoring patients that are at major risk of IA formation and growth. This reduced-order modelling approach allows to compute haemodynamic variables across large vessel networks [14] and was employed to investigate various aspects of cerebral flow [15], including its links with the CoW anatomical variations and occlusions [16], early vascular ageing [17], under hyperperfusion following vascular surgery [18, 19] and pathogenesis of cerebral small vessel disease [20]. Despite these efforts, blood flow changes across the whole CoW with an evolving aneurysm(s) are poorly documented.

Here, we present a computational pipeline that can be used for systematically quantifying longitudinal blood flow changes across the whole CoW in patients with different morphological features and investigating their link with aneurysm development. Furthermore, this approach may be applied in the future across large patient cohorts with specific characteristics, for identifying eventual common haemodynamic features and establishing new indexes.

## Materials and Methods

### Clinical Data Collection and Vascular Network Reconstruction

This study was approved by the UCLA Institutional Review Board. UCLA Medical Center’s clinical electronic records over the period of 2020–2021 were used. MRA showed to be highly effective in detecting diameter changes across the cerebral vasculature, and therefore it is commonly used for monitoring IA growth [21]. We selected patients with at least two follow-up imaging studies to classify the IA growth status (stable or growing). Aneurysm identification and classification were carried out as previously done by Chien et al. [22]. Patients’ longitudinal MRA consisting of an initial scan and a follow-up scan were used to reconstruct the patients’ vascular models. A total of seven patients with 2 scans each (total of 14 3D-MRA) were acquired. Table 1 summarizes aneurysm(s) information for each patient.

**Table 1:** Aneurysm information for the patient cohort. The reported patient age refers to the first scan

Patient	Age (years)	Sex	Aneurysm location(s)	Aneurysm size(s) (mm)	Evolution	Follow-up interval (years)
1	71	Female	R ICA G3, L PCoA	2.1, 4.1	stable, growing	4
2	69	Male	L PCoA	2.0	stable	6
3	41	Male	L ICA G3	3.0	stable	10
4	39	Female	L ICA G3, L MCA G1	6.8, 5.0	growing, growing	8
5	53	Male	ACoA	2.0	stable	7
6	53	Female	L ICA G3	2.0	stable	3
7	46	Female	R ICA G3	3.0	stable	10

**Fig. 1:**
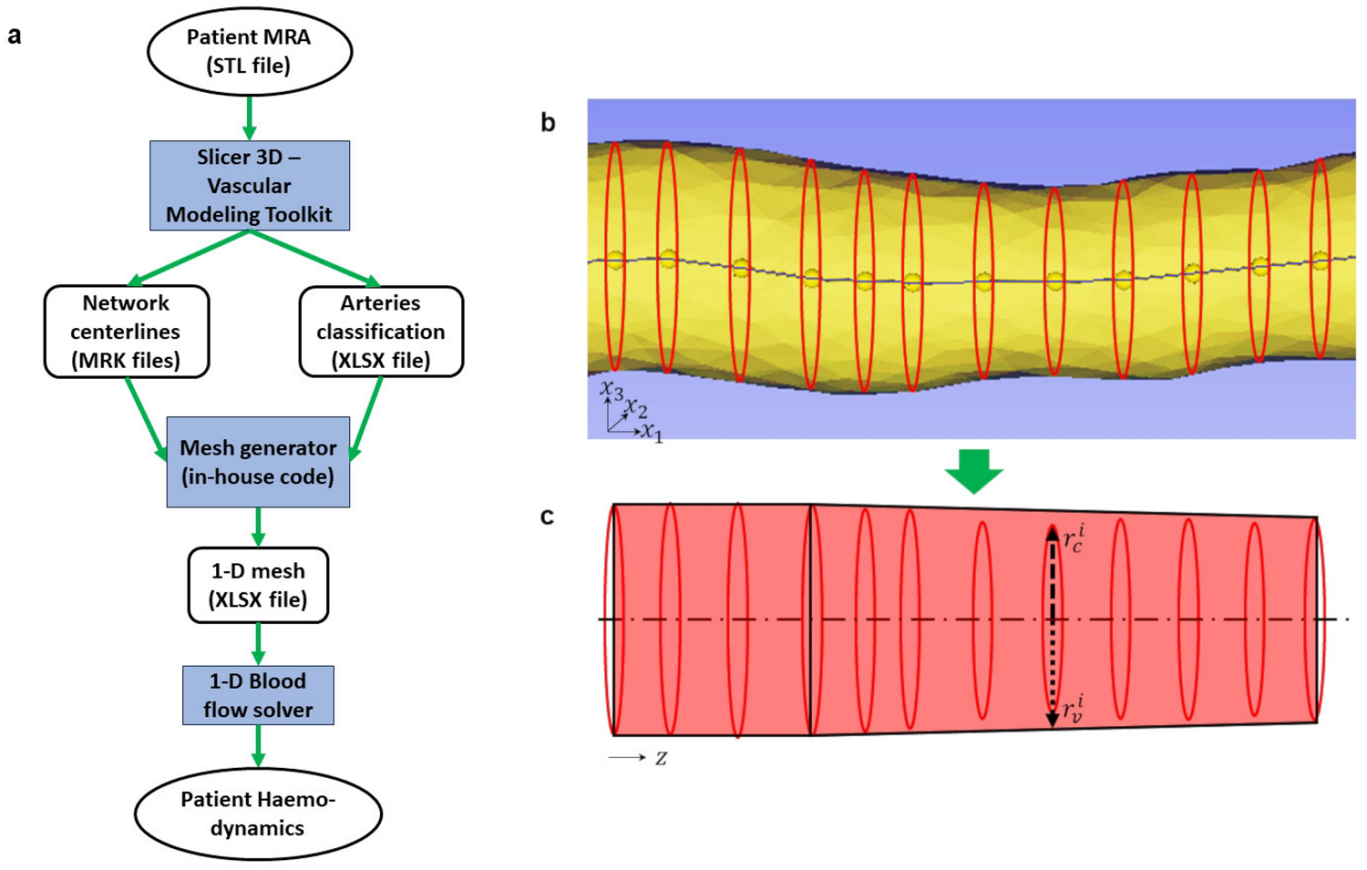
Semi-automated pipeline for computing patient haemodynamics across the cerebral circulation (**a**). Image segmentation process: segmented artery with centerline (**b**); vessels reconstructed by using some of the cross-sectional area along the centerline (**c**). $r_{{{c}}}^{i}$ is the radius associated to an $i^{{\text{th}}}$ centerline point whilst $r_{{{v}}}^{i}$ is the radius of the reconstructed smoothed vascular segment at the $i^{{\text{th}}}$ centerline point. Arteries classification is manually carried out by the user

**Fig. 2:**
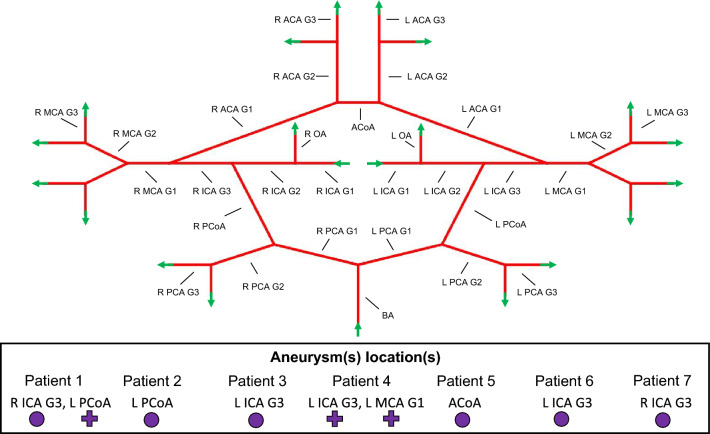
Reference CoW network with vessel labelling. Green arrows indicate inflow/outflow conditions at the boundaries. This scheme will be used for reporting blood flow distribution across the CoW and is not informative regarding the length nor the cross-sectional area (variable along the vessel axis) of the vessels. Locations of the aneurysm(s) for each patient are reported; stable aneurysms are indicated with purple full circles whilst growing aneurysms are indicated with purple full crosses

The methodology for deriving a 1-D (vessel network) mesh from a patient image is schematized in Fig. 1a. The software 3D Slicer 5.0.3—Vascular Modelling Toolkit was used (with default settings) for the automatic vessel segmentation and centreline extraction from the patient’s vascular image (STL file). As a result, each segmented artery was recorded in an MRK file as a series of not necessarily equidistant points located in space, with associated radii, forming a one-dimensional vessel with variable cross-sectional area. To minimize the amount of mesh data without significantly sacrificing the resolution, each segmented artery was iteratively re-subdivided into a finite number of shorter reconstructed vascular segments, with a cross-sectional area profile that can only vary in a monotonic way along the axial direction (Fig. 1b, c). The termination/accuracy of this process is controlled through a user-defined ‘smoothness coefficient’ which indicates the maximum relative error between the reconstructed and the original geometries per new vascular segment: $\gamma =\frac{\sqrt{\sum_{i=1}^{{n}_{c}}{\left(\frac{{r}_{c}^{i}-{r}_{v}^{i}}{{r}_{c}^{i}}\right)}^{2}}}{{n}_{c}}$ where ${n}_{c}$ is the number of considered centerline points of the new segment, ${r}_{c}^{i}$ with *i* = 1, …, ${n}_{c}$ is the radius associated to an ${i}^{th}$ centerline point whilst ${r}_{v}^{i}$ with *i* = 1, …, ${n}_{c}$ is the radius of the reconstructed smoothed vascular segment at the ${i}^{th}$ centerline point. The vascular network segmentation, together with the vessel connectivity matrix definition represent the key-steps for the generation of the 1-D mesh (saved as XLSX file) which are automatically carried out by means of one in-house Python.3 script. The overall process is considered semi-automatic as the user needs to manually label the arteries segmented through the software Slicer 3D—Vascular Modeling Toolkit (Fig. 1a). It is worth highlighting that, in this study, we define an ‘artery’ as the whole vessel linking two network branching nodes (or branching node and boundary node), which may be composed by different vascular segments. Here we label the arteries of the CoW as listed in Table 2 and shown in Fig. 2. Table 2 also reports the size of each vessel (axial length and mean radius) averaged across the patient cohort for Scan 1 and Scan 2. The vascular representation in Fig. 2, although not reporting some morphological details of the patient-specific reconstructed networks (such as arterial length, diameter and orientation), serves as a reference scheme for visualizing blood flow distribution across the cerebral circulation and making systematic comparisons.

**Table 2:** CoW vessel labelling and morphological data averaged across the 7 patients. Data is reported as mean ± standard deviation. Patient 6 does not present ACoA

Vessel	Abbreviation	Axial length Scan 1 (cm)	Radius Scan 1 (cm)	Axial length Scan 2 (cm)	Radius Scan 2 (cm)	Axial length relative difference between scans (−)	Radius relative difference between scans (−)
Right Internal Carotid Artery Generation 1	R ICA G1	5.26 ± 0.69	0.24 ± 0.02	5.2 ± 0.62	0.24 ± 0.02	− 0.01 ± 0.11	0.0 ± 0.02
Left Internal Carotid Artery Generation 1	L ICA G1	5.48 ± 0.86	0.24 ± 0.02	5.48 ± 0.6	0.25 ± 0.02	0.0 ± 0.07	0.03 ± 0.01
Right Internal Carotid Artery Generation 2	R ICA G2	0.88 ± 0.17	0.2 ± 0.02	0.98 ± 0.24	0.2 ± 0.03	0.09 ± 0.06	− 0.01 ± 0.04
Left Internal Carotid Artery Generation 2	L ICA G2	0.91 ± 0.18	0.21 ± 0.02	0.93 ± 0.2	0.21 ± 0.03	0.01 ± 0.06	− 0.0 ± 0.07
Right Internal Carotid Artery Generation 3	R ICA G3	0.65 ± 0.09	0.21 ± 0.02	0.63 ± 0.13	0.2 ± 0.03	− 0.05 ± 0.13	− 0.02 ± 0.06
Left Internal Carotid Artery Generation 3	L ICA G3	0.62 ± 0.18	0.23 ± 0.05	0.64 ± 0.18	0.21 ± 0.02	0.01 ± 0.15	− 0.09 ± 0.17
Right Ophthalmic Artery	R OA	0.82 ± 0.28	0.09 ± 0.03	0.72 ± 0.28	0.09 ± 0.03	− 0.22 ± 0.38	0.02 ± 0.19
Left Ophthalmic Artery	L OA	0.57 ± 0.07	0.09 ± 0.02	0.74 ± 0.29	0.09 ± 0.02	0.02 ± 0.62	− 0.0 ± 0.22
Basilar Artery	BA	3.04 ± 0.25	0.17 ± 0.01	3.1 ± 0.42	0.16 ± 0.02	0.01 ± 0.06	− 0.09 ± 0.07
Right Middle Cerebral Artery Generation 1	R MCA G1	2.27 ± 0.81	0.16 ± 0.01	2.32 ± 0.8	0.15 ± 0.01	0.03 ± 0.07	− 0.07 ± 0.07
Left Middle Cerebral Artery Generation 1	L MCA G1	2.51 ± 0.86	0.17 ± 0.02	2.54 ± 0.76	0.16 ± 0.01	0.03 ± 0.08	− 0.09 ± 0.14
Right Middle Cerebral Artery Generation 2	R MCA G2	2.93 ± 0.76	0.1 ± 0.02	3.07 ± 0.86	0.08 ± 0.01	0.04 ± 0.05	− 0.17 ± 0.22
Left Middle Cerebral Artery Generation 2	L MCA G2	2.61 ± 0.77	0.1 ± 0.01	2.72 ± 0.92	0.09 ± 0.01	0.03 ± 0.05	− 0.09 ± 0.17
Right Middle Cerebral Artery Generation 3	R MCA G3	1.44 ± 0.3	0.07 ± 0.01	1.4 ± 0.23	0.06 ± 0.01	− 0.05 ± 0.22	− 0.09 ± 0.27
Left Middle Cerebral Artery Generation 3	L MCA G3	1.48 ± 0.41	0.07 ± 0.01	1.5 ± 0.38	0.06 ± 0.01	0.0 ± 0.19	− 0.1 ± 0.17
Right Posterior Cerebral Artery Generation 1	R PCA G1	0.87 ± 0.18	0.13 ± 0.03	0.9 ± 0.18	0.11 ± 0.03	0.03 ± 0.08	− 0.21 ± 0.12
Left Posterior Cerebral Artery Generation 1	L PCA G1	0.87 ± 0.18	0.11 ± 0.03	0.88 ± 0.17	0.11 ± 0.03	0.01 ± 0.06	− 0.04 ± 0.14
Right Posterior Cerebral Artery Generation 2	R PCA G2	2.57 ± 0.37	0.11 ± 0.01	2.63 ± 0.42	0.09 ± 0.01	0.02 ± 0.03	− 0.28 ± 0.2
Left Posterior Cerebral Artery Generation 2	L PCA G2	2.8 ± 0.37	0.11 ± 0.01	2.88 ± 0.36	0.1 ± 0.01	0.03 ± 0.05	− 0.17 ± 0.16
Right Posterior Cerebral Artery Generation 3	R PCA G3	1.46 ± 0.32	0.08 ± 0.02	1.56 ± 0.21	0.07 ± 0.01	0.06 ± 0.21	− 0.25 ± 0.31
Left Posterior Cerebral Artery Generation 3	L PCA G3	1.35 ± 0.2	0.08 ± 0.01	1.34 ± 0.12	0.07 ± 0.02	− 0.01 ± 0.15	− 0.12 ± 0.21
Right Posterior Communicating Artery	R PCoA	1.46 ± 0.2	0.07 ± 0.02	1.53 ± 0.21	0.07 ± 0.03	0.04 ± 0.03	− 0.06 ± 0.35
Left Posterior Communicating Artery	L PCoA	1.49 ± 0.22	0.08 ± 0.03	1.53 ± 0.15	0.07 ± 0.04	0.03 ± 0.07	− 0.16 ± 0.35
Anterior Communicating Artery	ACoA	0.31 ± 0.12	0.14 ± 0.07	0.34 ± 0.13	0.12 ± 0.07	0.08 ± 0.12	− 0.64 ± 1.62
Right Anterior Cerebral Artery Generation 1	R ACA G1	2.17 ± 0.87	0.12 ± 0.03	2.16 ± 0.89	0.1 ± 0.03	− 0.01 ± 0.02	− 0.21 ± 0.22
Left Anterior Cerebral Artery Generation 1	L ACA G1	1.89 ± 0.84	0.12 ± 0.02	1.97 ± 0.84	0.11 ± 0.02	0.04 ± 0.07	− 0.13 ± 0.21
Right Anterior Cerebral Artery Generation 2	R ACA G2	3.66 ± 0.76	0.1 ± 0.01	3.39 ± 0.26	0.09 ± 0.02	− 0.08 ± 0.21	− 0.18 ± 0.25
Left Anterior Cerebral Artery Generation 2	L ACA G2	3.59 ± 0.6	0.1 ± 0.01	3.61 ± 0.47	0.09 ± 0.01	− 0.0 ± 0.15	− 0.23 ± 0.21
Right Anterior Cerebral Artery Generation 3	R ACA G3	1.08 ± 0.19	0.08 ± 0.01	0.86 ± 0.29	0.07 ± 0.02	− 0.41 ± 0.28	− 0.34 ± 0.53
Left Anterior Cerebral Artery Generation 3	L ACA G3	1.19 ± 0.37	0.08 ± 0.02	0.83 ± 0.16	0.07 ± 0.01	− 0.46 ± 0.31	− 0.18 ± 0.33

### Blood Flow Modelling

#### Governing Equations

Blood flow through cerebral arteries was modelled as Newtonian and incompressible, in line with key reference works [16, 20]. The governing equations are the mass and momentum conservation along the axial direction, combined with a material law describing how the vascular wall deforms upon normal loading. The hydrostatic pressure (*P*) and volumetric flow (*Q*) are described as follows [23, 24]:

1 $$C_{A} \frac{\partial P}{{\partial t}} + \frac{\partial Q}{{\partial z}} = 0,$$

2 $$\frac{\rho }{A}\frac{\partial Q}{{\partial t}} + \frac{\rho }{A}\frac{\partial }{\partial z}\left( {\frac{{Q^{2} }}{A}} \right) + \frac{\partial P}{{\partial z}} + 8\pi \mu \frac{Q}{{A^{2} }} = 0,$$

3 $$P = P_{\text{ext}} + P_{0} + \frac{\beta }{{A_{0} }}\left( {\sqrt A - \sqrt {A_{0} } } \right),$$

where *C*_*A*_ = $\frac{\partial A}{\partial P}$ is the vessel wall compliance, *A* is the cross-sectional area of the vessel, *ρ* is the blood density, *μ* is the blood dynamic viscosity, ${P}_{\text{ext}}$ is the external pressure applied on the vascular wall, ${P}_{0}$ is the reference pressure, *A*_*0*_ is the reference area, whilst β is the vessel wall elasticity parameter, which is calculated as

4 $$\beta = \frac{4}{3}\sqrt \pi Eh,$$

where *E* and *h* are the Young’s modulus and the thickness of the vascular wall, respectively.

#### Boundary Conditions

The blood flow conditions at the network inlets were prescribed following the study by Cebral et al. [25]. These authors derived relationships from in-vivo measurements that describe how the maximum inflow ${\widehat{Q}}_{in}$ and the mean (in time/over a cardiac cycle) inflow ${\overline{Q} }_{in}$ in an artery relate to its spatially averaged reference cross-sectional area ${\overline{A} }_{0}$ (evaluated by averaging the nodal cross-sectional values of the inlet artery):

5 $$\hat{Q}_{in} = 70.80{ }\overline{A}_{0}^{1.7} ,$$

6 $$\overline{Q}_{in} = 48.21 \overline{A}_{0}^{1.84} .$$

Here for the boundary conditions at internal carotid and basilar artery level (R ICA G1, L ICA G1, BA) we defined time-dependent inflow signals that satisfy both (5) and (6). More information on the prescription of the inflow conditions is reported in the (Online) Supplementary Information file. We also introduced the carotid inflow ratio (θ) as the mean flow entering the right internal carotid (R ICA G1) over the mean inflow at the left internal carotid (L ICA G1), which can inform on eventual changes in the symmetry of flow across the CoW.

Outflow conditions at each network outlet were imposed using a 3-element Windkessel model representing the flow resistance and compliance of the downstream circulation. In this study the terminal coefficients (terminal resistance ${R}_{t}$ and terminal compliance ${C}_{t}$) for each cerebral downstream sub-region (anterior, middle, posterior and ophthalmic branching) as reported by Reymond et al. [26] were used. For the ophthalmic circulation we assumed values as found earlier [27], whilst the terminal flow resistance coefficient was redistributed as 20–80 (%) between the impedance and distal resistance coefficients.

#### Accounting for Imaging Resolution Level

The imaging resolution level may change from scan to scan due to various factors (i.e., type of scan, ability of the operator, environmental conditions). Since none of the recruited patients exhibited detectable changes in terms of lifestyle and cognitive capabilities over the considered time span, we assumed that their brain metabolic demand and associated requested blood supply were unchanged between scans. This allowed us to introduce a resolution scaling factor *α* for each patient, ensuring that the total averaged inflow in the CoW is the same between scans. This new parameter indicates whether the reference cross-sectional areas of the vascular network obtained from the follow-up scan (Scan 2) needs to be dilated (*α* > 1) or contracted (*α* < 1). To identify this scaling parameter for each patient we impose volumetric inflow equality between Scan 1 (indicated with *sc1*) and Scan 2 (indicated with *sc2*) combined with Eq. (6):

7 $$\left( {\overline{A}_{0,R ICA G1}^{ sc1} } \right)^{1.84} + \left( {\overline{A}_{0, L ICA G1}^{ sc1} } \right)^{1.84} + \left( {\overline{A}_{0, BA}^{sc1} } \right)^{1.84} = \left( {\alpha \overline{A}_{0, R ICA G1}^{ sc2} } \right)^{1.84} + \left( {\alpha \overline{A}_{0, L ICA G1}^{ sc2} } \right)^{1.84} + \left( {\alpha \overline{A}_{0, BA}^{ sc2} } \right)^{1.84},$$

where subscripts and superscripts are used for indicating the type of vessel and scan. Hence, we can evaluate $\alpha$ as

8 $$\alpha = \left( {\frac{{\left( {\overline{A}_{0, R ICA G1}^{ sc1} } \right)^{1.84} + \left( {\overline{A}_{0, L ICA G1}^{ sc1} } \right)^{1.84} + \left( {\overline{A}_{0,BA}^{ sc1} } \right)^{1.84} }}{{\left( {\overline{A}_{0, R ICA G1}^{ sc2} } \right)^{1.84} + \left( {\overline{A}_{0, L ICA G1}^{ sc2} } \right)^{1.84} + \left( {\overline{A}_{0, BA}^{sc2} } \right)^{1.84} }}} \right)^{{\frac{1}{1.84}}}.$$

Once identified, this patient-specific scaling factor was used for dilating/contracting all the cross-sectional areas ${A}_{0}$ (to be multiplied by *α*) of the vascular network obtained from Scan 2.

#### Numerical Procedure and Settings

Equations (1–3) were discretized in time using a second-order backward difference scheme and in space by means of an established finite volume scheme by Carson and Van Loon [23]. Lagrange multipliers were used to ensure continuity of mass and momentum at branching points such as bifurcations/trifurcations and between vascular segments of the same artery. Within this framework, blood flow variables *P, Q* and* A* were directly computed in time across the one-dimensional fluid network.

For all patients’ simulations:

- Each vascular segment was discretised into the lowest number of finite elements provided that the element size was equal or less than 1 mm, whilst the time step was fixed to 2e−4s.
- Ten cardiac cycles of the ‘inflow’ signals were simulated, which was enough to reach perfectly periodic solutions. The last two periods are then considered for the analysis.
- Blood density and dynamic viscosity were set equal to 1.04 g/cm^3^ and 0.04 poise, respectively.
- Intracranial venous and external pressures in the Windkessel model were set equal to 10 and 15 mmHg (in line with [16, 26]), respectively.
- Some vascular geometric and constitutive properties in Table 3 were set in line with values from literature [16, 26].
- The reference pressure ${P}_{0}$ was distributed across the network by considering typical mean pressure levels [20]: ${P}_{0}$=100 mmHg for the large inlet arteries (R/L ICA G1-G3, BA and R/L OA) whilst ${P}_{0}$=90 mmHg for the other smaller ones (Table 3).

**Table 3:** Vascular parameters adopted across the patient cohort. Thickness and Young's modulus are from [9], whilst peripheral vascular coefficients are from [16].

Vessel(s)	Thickness *h* (cm)	Elastic modulus *E* (1e6 Pa)	Elastic coefficient *β* (Pa*cm)	Reference pressure ${P}_{0}$ (mmHg)	Peripheral resistance ${R}_{t}$ (1e3 Pa s cm^-3^)	Peripheral compliance ${C}_{t}$ (1e−4 cm^3^ Pa^-1^)
R/L ICA G1-G3	0.05	0.8	94529.48	100.0	−	−
R/L MCA G1-G2	0.036	1.6	136122.4	90.0	−	−
R/L MCA G3	0.036	1.6	136122.4	90.0	10.0258144	0.0021
R/L OA	0.009375	1.6	35448.55	100.0	26.7710576	0.0003
R/L ACA G1-G2	0.03	1.6	113435.4	90.0	−	−
R/L ACA G3	0.03	1.6	113435.4	90.0	10.732421	0.00353
R/L PCoA	0.018	1.6	68061.22	90.0	−	−
BA	0.04	1.6	151247.2	100.0	−	−
ACoA	0.019	1.6	71842.4	90.0	−	−
R/L PCA G1-G2	0.027	1.6	102091.8	90.0	−	−
R/L PCA G3	0.027	1.6	102091.8	90.0	10.732421	0.00435

## Results

### Impact of Geometry Reconstruction on Patient-Specific Haemodynamics

First, we analysed the role of the geometric reconstruction accuracy on the simulated blood flow in the CoW for different patients. As anticipated above, the smoothness coefficient γ reflects the accuracy with which the vascular image is translated into the 1-D mesh, and it is implicitly associated with the number of vascular segments that compose the network. Since Lagrange multipliers are used for ensuring mass and momentum conservation between vascular segments, increasing the smoothness level means augmenting the size of the system of equations to be solved. Therefore, we aimed to identify a smoothness level that could represent a suitable trade-off between model geometric fidelity and computational efficiency. We considered Patients 1 and 5 as representative cases since they present profoundly different clinical situations, as Patient 1 has two aneurysms located at the R ICA G3 (stable) and L PCoA (growing) whilst the Patient 5 has one stable aneurysm located at the ACoA. In the following we indicate with $\overline{A }$ the mean cross-sectional area of a vessel obtained by averaging its nodal values in time (over one cardiac cycle) and in space (along the vessel length). In the same way $\overline{Q }$ and $\overline{P }$ represent the mean flow and pressure of the vessel, respectively. For these patients, haemodynamic simulations across the CoW were carried out by considering six levels of smoothness, namely *γ* = 0.005, 0.01, 0.02, 0.03, 0.04 and 0.05.

To identify a suitable smoothness level to be used throughout the study we evaluated, for each vessel, the relative difference in mean cross-sectional area obtained for *γ* = 0.01, 0.02, 0.03, 0.04 and 0.05 with respect to the case with the lowest smoothness coefficient (*γ* = 0.005, which is considered the reference case). For each *γ* we then computed a weighted average between the absolute values of the relative differences across all vessels in the network ${\varepsilon }_{net}^{\gamma }$. We used as weights the vessel lengths normalized with respect to the total network length. The network relative differences ${\varepsilon }_{net}^{\gamma }$ for Patients 1 and 5 are reported in Fig. 2 of the (Online) Supplementary Information file. These results indicate that, with *γ* = 0.01, the relative change in cross-sectional area (with respect to the case with *γ* = 0.005) is equal or lower than 0.2%, which can be considered more than acceptable. Hence, we used *γ* = 0.01 for the remaining simulations reported in this study.

### Quantification of Patient-Specific Blood Flow Changes Between Scans

**Fig. 3:**
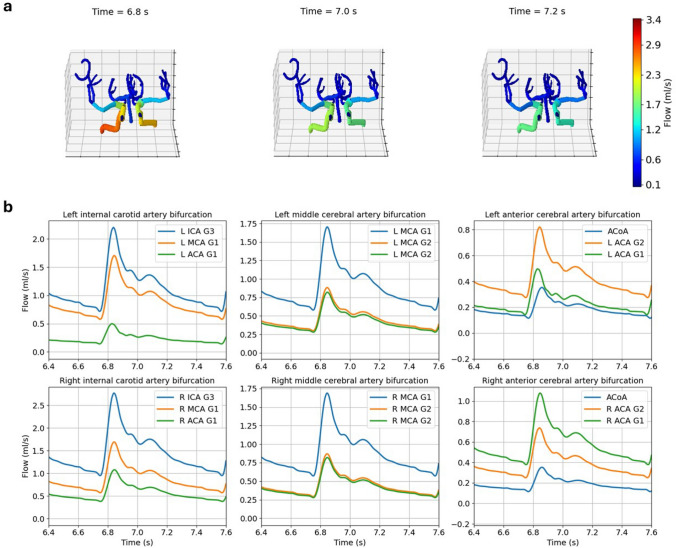
Blood flow distribution for Patient 1, Scan 1. Snapshots of flow distribution across the whole CoW at three different time points (**a**). Blood flow in time at different locations of the patient network (**b**)

**Fig. 4:**
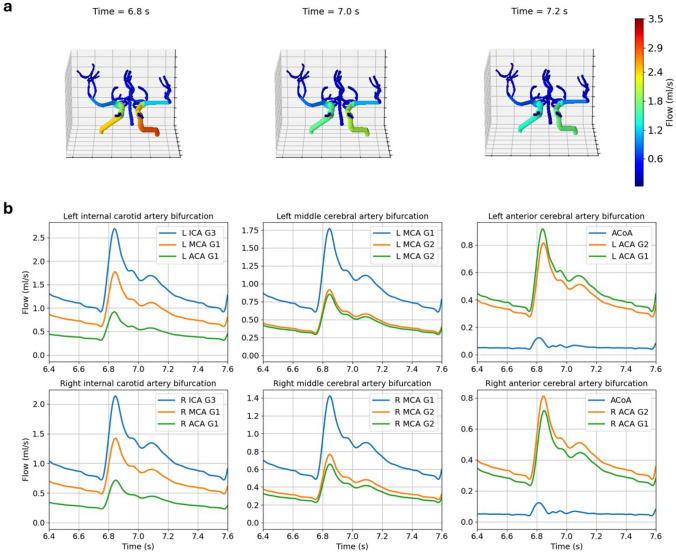
Blood flow distribution for Patient 1, Scan 2. Snapshots of flow distribution across the whole CoW at three different time points (**a**). Blood flow in time at different locations of the patient network (**b**)

The main use of the proposed computational pipeline is the quantification of patient longitudinal blood flow changes in the CoW. Patient 1 flow distribution at different time points for Scan 1 and 2 is reported in Figs. 3 and 4, respectively. The same figures also show how blood flow changes over a cardiac beat at six different locations of the CoW. Comparison between these figures indicates a change in role for the ACoA, which experienced a significant reduction in flow between Scan 1 and Scan 2. For this patient we observed that, interestingly, the carotid inflow ratio (*θ* = $\frac{{\overline{Q} }_{R ICA G1}}{{\overline{Q} }_{L ICA G1}}$) significantly changed between scans (*θ* = 1.15 for Scan 1 and *θ* = 0.84 for Scan 2), indicating that inflow dominance shifted from right to left internal carotid.

Figures 3 and 4 in the (Online) Supplementary Information file report the flow distribution over a cardiac cycle for the two scans of Patient 5. In this case we also observed a dramatic change in flow at the ACoA, where however the aneurysm remained stable. At this location the mean flow was almost zero in Scan 1, whilst after (in Scan 2) the blood flow was exclusively received from the L ACA G1. This highlights the key role of ACoA in the redistribution/balance of blood flow across the CoW despite morphological adaptations. Given the limited number of analysed patients we were not able to establish any correlation between flow changes and vascular locations. However, the above-reported results indicated that remarkable flow changes can happen at the ACoA, even if the aneurysm is developing in another vessel (as for Patient 1). For Patient 4 we recorded a significant inflow asymmetry at Scan 1 (*θ* = 1.15) which is almost balanced out at Scan 2 (*θ* = 1.01). We did not record significant changes in carotid inflow dominance for other four patients (Patient 2: *θ* = 0.74 for Scan 1 and *θ* = 0.68 for Scan 2, Patient 5: *θ* = 0.77 for Scan 1 and *θ* = 0.67 for Scan 2, Patient 6: *θ* = 0.96 for Scan 1 and *θ* = 0.80 for Scan 2, Patient 7: *θ* = 0.69 for Scan 1 and *θ* = 0.59 for Scan 2), while the opposite behaviour (inflow dominance shifted from left to right internal carotid) was observed for Patient 3 (*θ* = 0.96 for Scan 1 and *θ* = 1.15 for Scan 2). Altogether, these results indicate that the changes in inflow distribution over time exhibit extreme variability, which is likely dependent on different levels of vascular adaptation across the CoW.

**Fig. 5:**
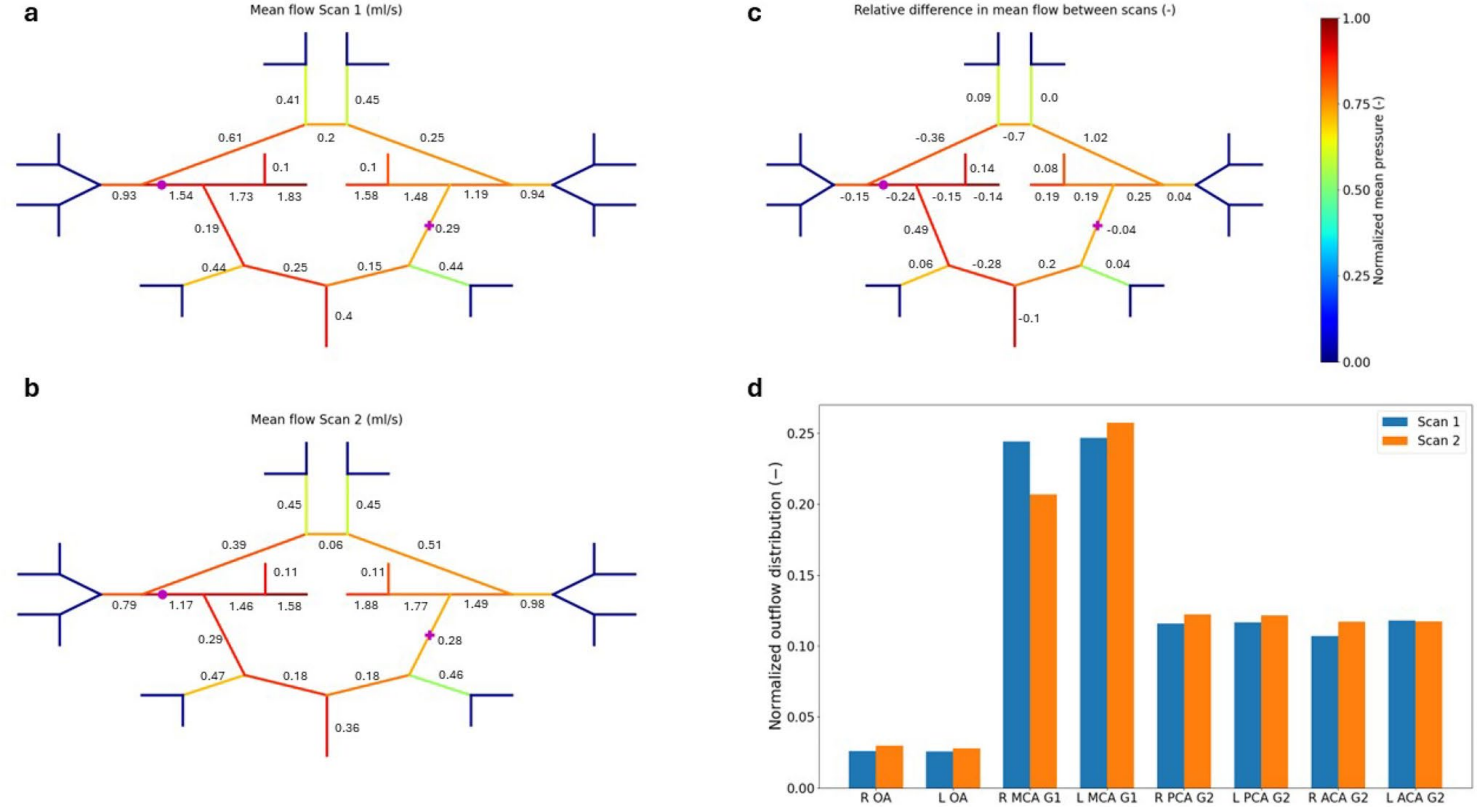
Normalized blood flow distribution across the CoW of Patient 1. Computed average blood flow in the vascular network derived from Scan 1 (**a**). Computed average blood flow in the vascular network derived from Scan 2 (**b**). Relative variation in average blood flow between scans (**c**). Normalized average blood flow distribution among outlets (**d**). The vessels are colored according to their mean pressure level. The aneurysm locations are indicated with purple full circles (stable) and full cross (growing). Normalized mean pressure is evaluated by considering all the vessels of the network as ${\overline{{P}} }_{{\text{norm}}}=(\overline{{{P}} }-{\text{min}}(\overline{{{P}} }))/({\text{max}}(\overline{{P} })-{\text{min}}(\overline{{P} }))$

In this study we are especially interested in the relative variation of mean flow ($\overline{Q }$) between scans, which at vessel level is evaluated as ${\varepsilon }^{\text{sc}}=\frac{{\overline{Q} }^{sc2}-{\overline{Q} }^{sc1}}{{\overline{Q} }^{sc1}}$. For Patient 1, with one aneurysm classified as ‘growing’, we observed some significant flow changes in some vascular beds (Fig. 5). A significant flow increment was recorded in the L ACA G1 and R PCoA and decrease in ACoA, which may indicate a change in the functionality of these vascular beds. We also evaluated how the total blood flow entering the cerebral circulation is distributed between subregions. Interestingly, the presence of a stable aneurysm at the end of the right carotid artery was associated with a reduction (with respect to Scan 1) in outflow through the right middle cerebral artery whilst the outflow near the growing aneurysm (L PCA) did not significantly change.

Figure 5 of the (Online) Supplementary Information file reports the blood flow changes experienced by Patient 5. Although this patient’s aneurysm was classified as stable, our results suggest that significant haemodynamic changes occurred over time across the CoW. Alongside the previously reported ACoA mean flow increase (+2973%), substantial flow changes also occurred along the R PCoA (+509%), which highlights its physiological role. We speculate that these arteries were ‘recruited’ to compensate other vascular changes in the CoW. This reinforces the need for further investigating the link between aneurysm development and the CoW haemodynamics.

**Fig. 6:**
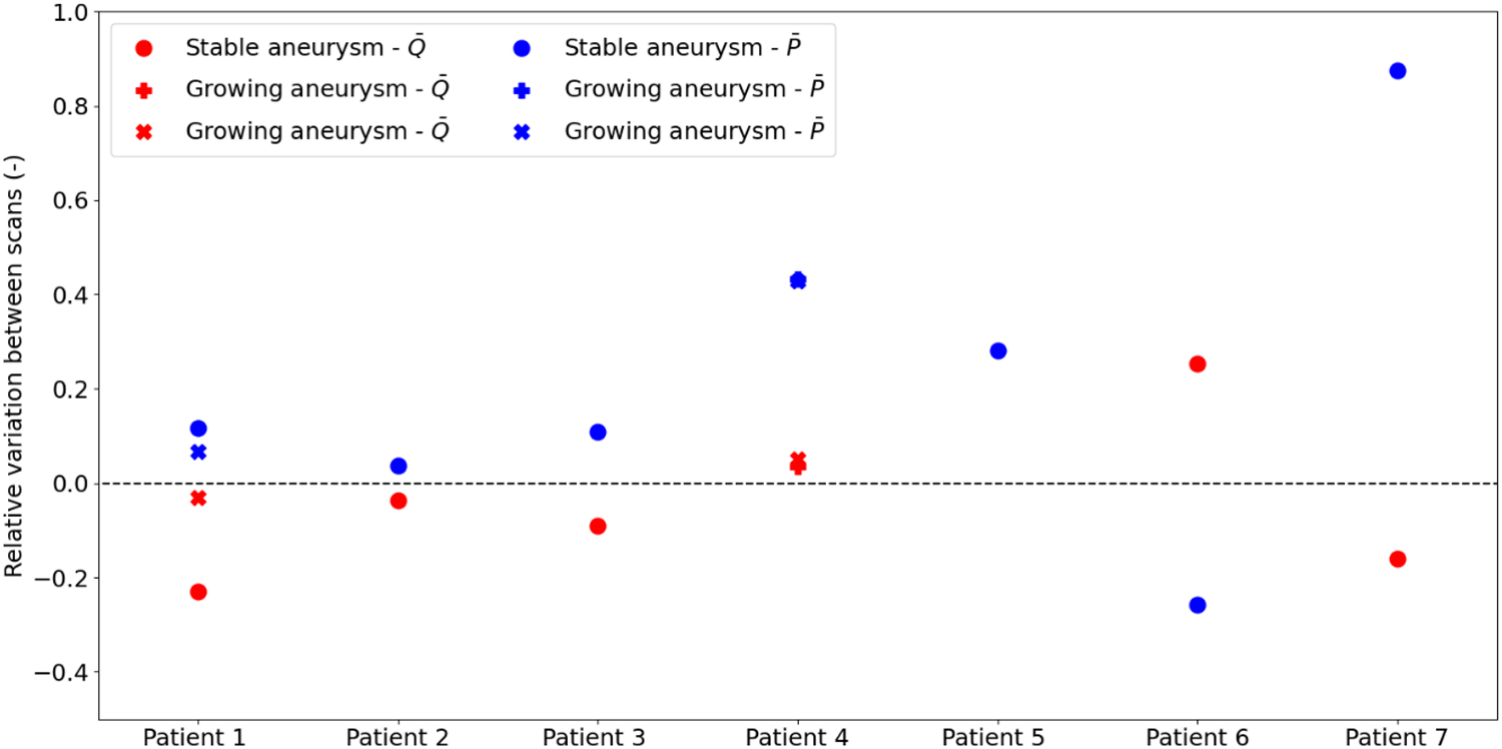
Relative variation of mean flow and pressure at the vessel with aneurysm across the patient cohort. The relative variation in mean flow for Patient 5 is 29.73

Now we consider how flow changes within the vessel presenting the aneurysm across the whole patient cohort (Fig. 6). In four cases (Patients 1, 2, 3 and 7), the mean flow in the vessel with aneurysm decreased between scans. Interestingly, also growing aneurysms (Patients 1 and 4) were associated with moderate flow changes over time, whilst the opposite situation only occurred for one of the stable aneurysms (Patient 5). In terms of mean pressure variation, only Patients 6 reported a decrease between Scan 1 and Scan 2. For all other patients, mean pressure increased over time. Very similar results were obtained in terms of relative variation (between scans) of peak flow ($\widehat{Q}$) and peak pressure ($\widehat{P}$) recorded in the vessel with aneurysm (Fig. 6 in the Supplementary Information file).

### Sensitivity of Flow Results to Imaging Resolution Level, Outflow Resistance and Vascular Compliance

Here we investigated the effect of some simulation settings on the patient results. In the following, for each patient, the relative flow variation (in the vessel with aneurysm) between scans reported in "Quantification of patient-specific blood flow changes between scans" section is taken as a reference (and indicated with ${\varepsilon }_{\text{ref}}^{\text{sc}}$). We considered:

- one case (indicated as ‘*α* = 1’) in which the vascular mesh from Scan 2 is not scaled for satisfying inflow equality between scans and therefore *α* = 1;
- two cases (indicated as ‘0.5*R*_*t*_’ and ‘2*R*_*t*_’, respectively) in which the total terminal flow resistances for all the cerebral sub-regions are increased/decreased (+100%/-100%) with respect to the values reported in Table 3;
- two cases (indicated as ‘0.5*E*’ and ‘2*E*’, respectively) in which the elastic modulus (and therefore vascular compliance) for all the cerebral arteries is increased/decreased (+100%/-100%) with respect to the values reported in Table 3.

For each of these cases, we report the relative error of the (relative) flow variation with respect to the reference case ($\eta =\frac{{\varepsilon }_{i}^{\text{sc}}-{\varepsilon }_{\text{ref}}^{\text{sc}}}{{\varepsilon }_{\text{ref}}^{\text{sc}}}$ with ${\varepsilon }_{i}^{\text{sc}}$ the relative flow variation between scans for the *i*th case). Table 4 summarizes the simulation results for each case across all patients.

**Table 4:**
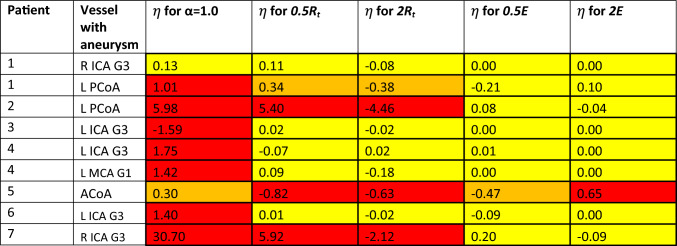
Effect of imaging resolution level (*α*), terminal flow resistance (*R*_t_) and elastic modulus (*E*) on the relative error of the (relative) flow variation between scans with respect to the reference case *η* (−). Colour code: red for |*η*| > 0.5, orange for 0.25 ≤ |*η*| ≤ 0.5, yellow for |*η*| < 0.25

This data indicates that scaling the mesh from Scan 2 to ensure the same inflow (see procedure in "Accounting for imaging resolution level" section) has a profound impact on the results of all patients. Interestingly, the parameter representing the downstream flow resistance played an important role only for some of the patients (Patients 1, 2, 5 and 7). On the other hand, changing vascular compliance across the vessel network seems to have, for most of the patients (with the exception of Patient 5), a much more limited effect on blood flow changes between scans.

## Discussion

In clinical neuroradiology diagnosis, follow-up neuroimaging is critical in the monitoring of cerebrovascular disease. It is used to observe whether an aneurysm is stable or growing but it is also instrumental for the discovery of *de novo* aneurysm formation as the whole CoW may undergo significant changes over time. In this work we proposed, for the first time, a computational pipeline to investigate longitudinal flow changes in response to vascular adaptations, with the aim of elucidating the hemodynamic influence of the whole CoW on aneurysm evolution. As a pilot study, we applied this methodology to a patient cohort with vasculatures exhibiting different anatomical features and aneurysm growth status. Firstly, we identified which smoothness/geometric reconstruction coefficient is necessary for obtaining reasonably accurate predictions. Then, we systematically reported changes in flow distribution between scans for a selected number of patients. We also compared the relative variation in mean (peak) flow and pressure at the vessel with the aneurysm across the seven patients. Interestingly, growing aneurysms were associated with limited local haemodynamic changes whilst the opposite was recorded for one stable IA (Patient 5). Although the low number of patients did not allow us to identify conclusive correlations between haemodynamic features and aneurysm development, we observed significant haemodynamic changes at vessels far from the aneurysm location, suggesting vascular adaptations occur at a systemic level. Our preliminary results indicate that varying the Young’s modulus in the adopted tube law does not have, overall, a significant impact on the blood flow changes between scans. On the other hand, the level of imaging resolution (represented through *α*) had a huge impact on the predicted flow variation between scans. This is a relevant finding because many image-based modelling studies do not recalibrate the segmented mesh for longitudinal comparison. Given the strong link between inflow and cross-sectional area at carotid and basilar level, the omission of this element during the mesh reconstruction may lead to significantly different haemodynamic predictions, and therefore requires urgent clarification. We recorded high variability in the inflow distribution (represented by the parameter *θ*) changes over time and this aspect also deserves further investigation. Interestingly, terminal flow resistance *R*_*t*_ (associated with outflow conditions) has a less defined role on the longitudinal flow variation estimation, as it significantly impacts only half of the patient cohort. To shed the light on the role of downstream vascular territories, the current platform could be used in conjunction with in vitro models [28, 29] and/or in vivo recordings of pressure and flow at different CoW locations [30].

The proposed pipeline presents some limitations that pave the way for potential future extensions. Since the current methodology is developed for quantifying longitudinal flow changes across the whole CoW, each aneurysm was treated as an axisymmetric wall enlargement/deformation. To improve the predictive capacity of the framework one could couple the 1-D vessel network with a high-fidelity sub-model able to accurately represent the local aneurysm haemodynamics and associated wall deformation. The computational cost associated with such methodology could be however prohibitive and not applicable to clinical settings. For this reason, we are currently exploring the integration into our framework of alternative reduced-order models (such as [31]) which can capture more in detail the local flow-aneurysm interaction. Moreover, this modelling framework could be enhanced further by integrating tube laws that can better capture the vascular structural response [2, 32] and adaptation processes [24, 33–35]. In this way, classical Growth&Remodelling models [33, 35] for vascular adaptation could be extended to large vascular networks and validated by considering the time evolution of blood flow conditions in the aneurysm and its neighbouring vessels. This work represents therefore a valid basis for future longitudinal investigations targeting the systematic characterization of the link between aneurysm development and cerebral flow conditions.

### Supplementary Information

Below is the link to the electronic supplementary material.

Supplementary file1 (PDF 1663 kb)
