# Supplementary material for: A Computational Pipeline to Investigate Longitudinal Blood Flow Changes in the Circle of Willis of Patients with Stable and Growing Aneurysms
Source: Ann Biomed Eng. 2024 Apr 14;52(8):2000–12. doi: 10.1007/s10439-024-03493-1 (PMC11247057; doi:10.1007/s10439-024-03493-1)
Supplement: Supplementary file 1 — Supplementary file1 (PDF 1663 kb) [file 10439_2024_3493_MOESM1_ESM.pdf]

Supplementary Information

A computational pipeline to investigate longitudinal blood flow changes in the Circle of Willis of patients with stable and growing aneurysms

Alberto Coccarelli*, Raoul van Loon*# and Aichi Chien**

*Zienkiewicz Institute for Modelling, Data and AI, Faculty of Science and Engineering, Swansea University, UK

#Biomedical Engineering Simulation and Testing Lab, Department of Biomedical Engineering, Swansea University, UK

** Radiological Sciences, School of Medicine, University of California Los Angeles, USA

Corresponding author: Alberto Coccarelli, Zienkiewicz Institute for Modelling, Data and AI, Faculty of Science and Engineering, Swansea University, UK. Email: alberto.coccarelli@swansea.ac.uk

Inflow boundary conditions prescription

For each patient's inlet vessel, the average reference area $\bar{A}_0$ is used for computing the maximum inlet flow rate $\hat{Q}_{in}$ and the mean inlet flow rate $\bar{Q}_{in}$ (see equations in the Manuscript).

The time-dependent inflow signal over a cardiac cycle *T* can generally be described as:

$Q_{in}(t) = c_0 + c_1 f_{in}^0(t),$ (1)

where c_0 and c_1 are re-scaling parameters to be defined according to $\hat{Q}_{in}$ with $\bar{Q}_{in}$, whilst $f_{in}(t)$ ϵ [0,*T*) is a time-dependent signal (see Figure 1) for which $\int_0^T f_{in}(t)dt = 1$.

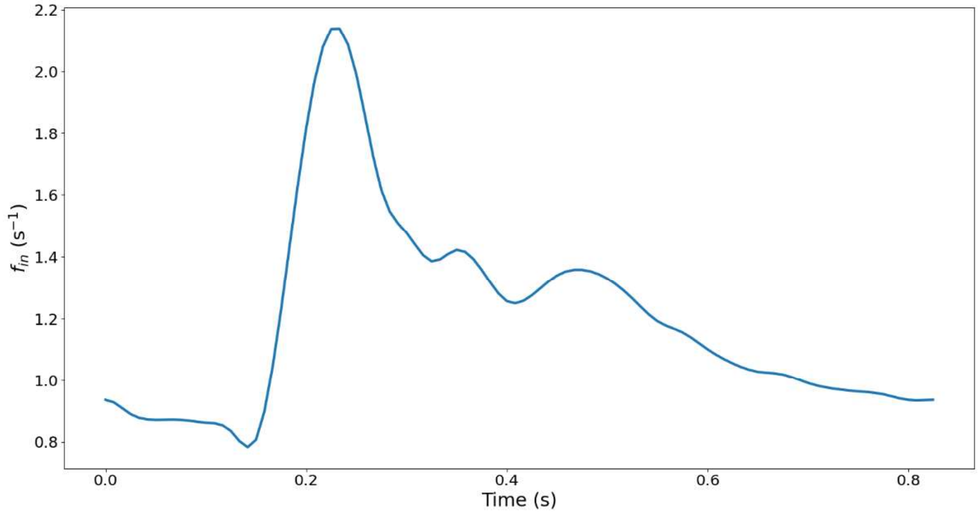

Figure 1 Time-dependent signal $f_{in}(t)$ *over a cardiac cycle.*

Given $\hat{Q}_{in}$ and $\bar{Q}_{in}$, the inflow signal needs to satisfy the following conditions:

$$max\big(Q_{in}(t)\big) = \hat{Q}_{in} \quad \text{and} \quad \frac{1}{T}\int_0^T Q_{in}(t)dt = \bar{Q}_{in}. \quad (2)$$

After few steps, the re-scaling parameters c_0 and c_1 can be defined as:

$$c_0 = \hat{Q}_{in} - \big(\hat{Q}_{in} - \bar{Q}_{in}\big)\frac{f_{in}^{max}T}{f_{in}^{max}T-1} \quad \text{and} \quad c_1 = \frac{(\hat{Q}_{in} - \bar{Q}_{in})T}{f_{in}^{max}T-1}, \quad (3)$$

where $f_{in}^{max} = \max(f_{in})$.

Effect of geometric reconstruction accuracy on simulation results

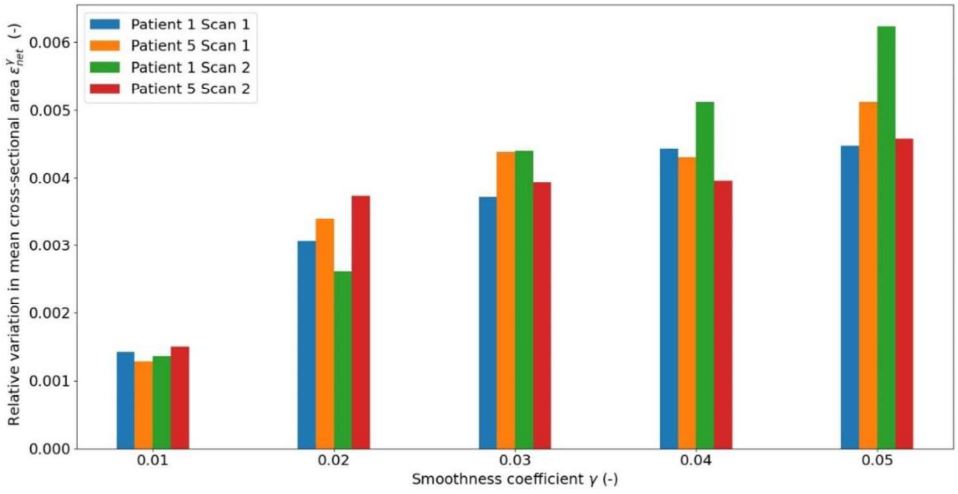

Figure 2 Effect of smoothness coefficient (γ=0.01, 0.02, 0.03, 0.04, 0.05) on mean cross-sectional area relative variation for Patients 1 and 5, Scans 1 and 2. Relative variations are calculated by considering reference values obtained with a smoothness coefficient equal to 0.005.

Longitudinal blood flow changes in Patient 5

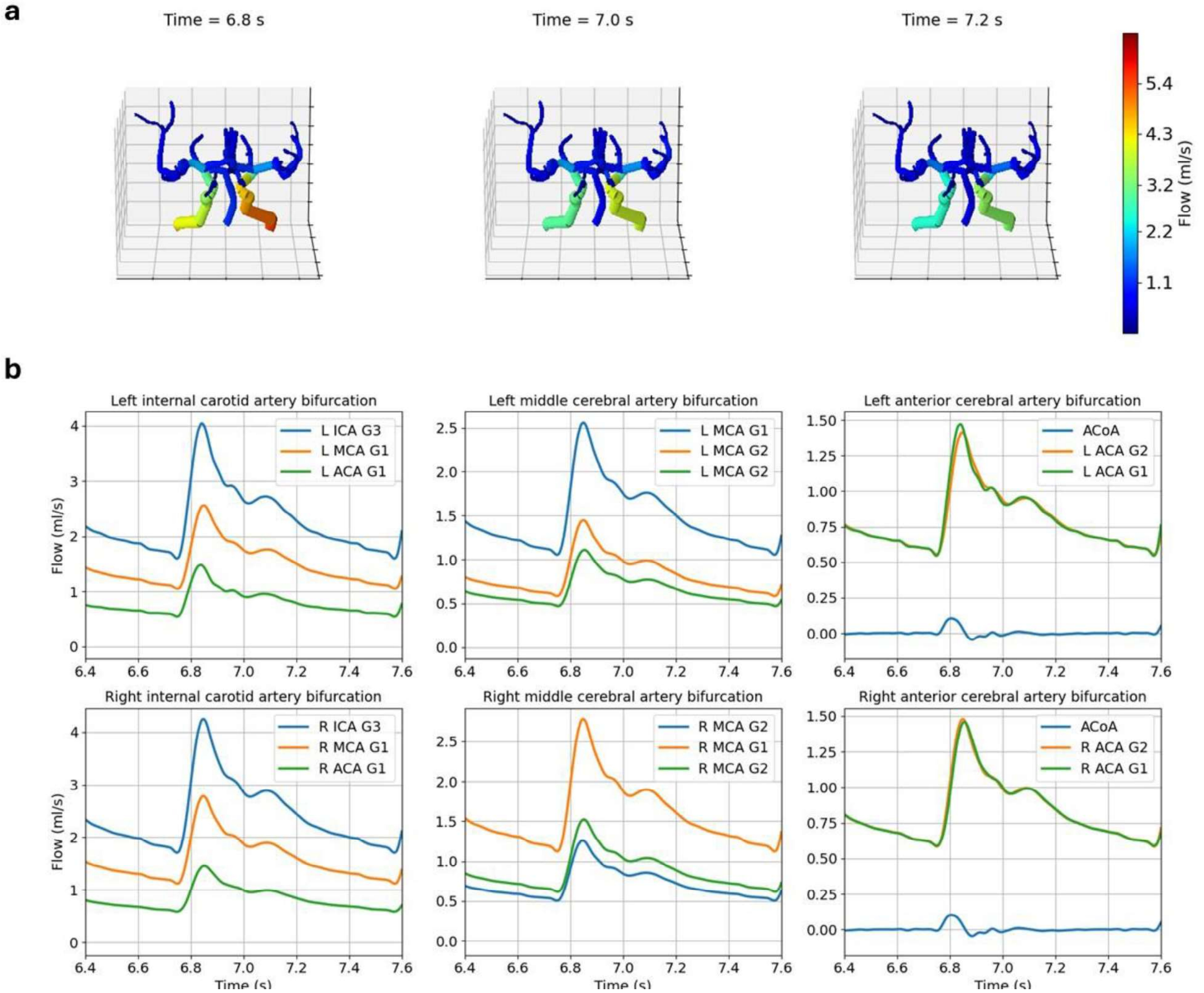

Figure 3 Blood flow distribution for Patient 5, Scan 1. Snapshots of flow distribution across the whole CoW at three different time points (a). Blood flow in time at different locations of the patient network (b).

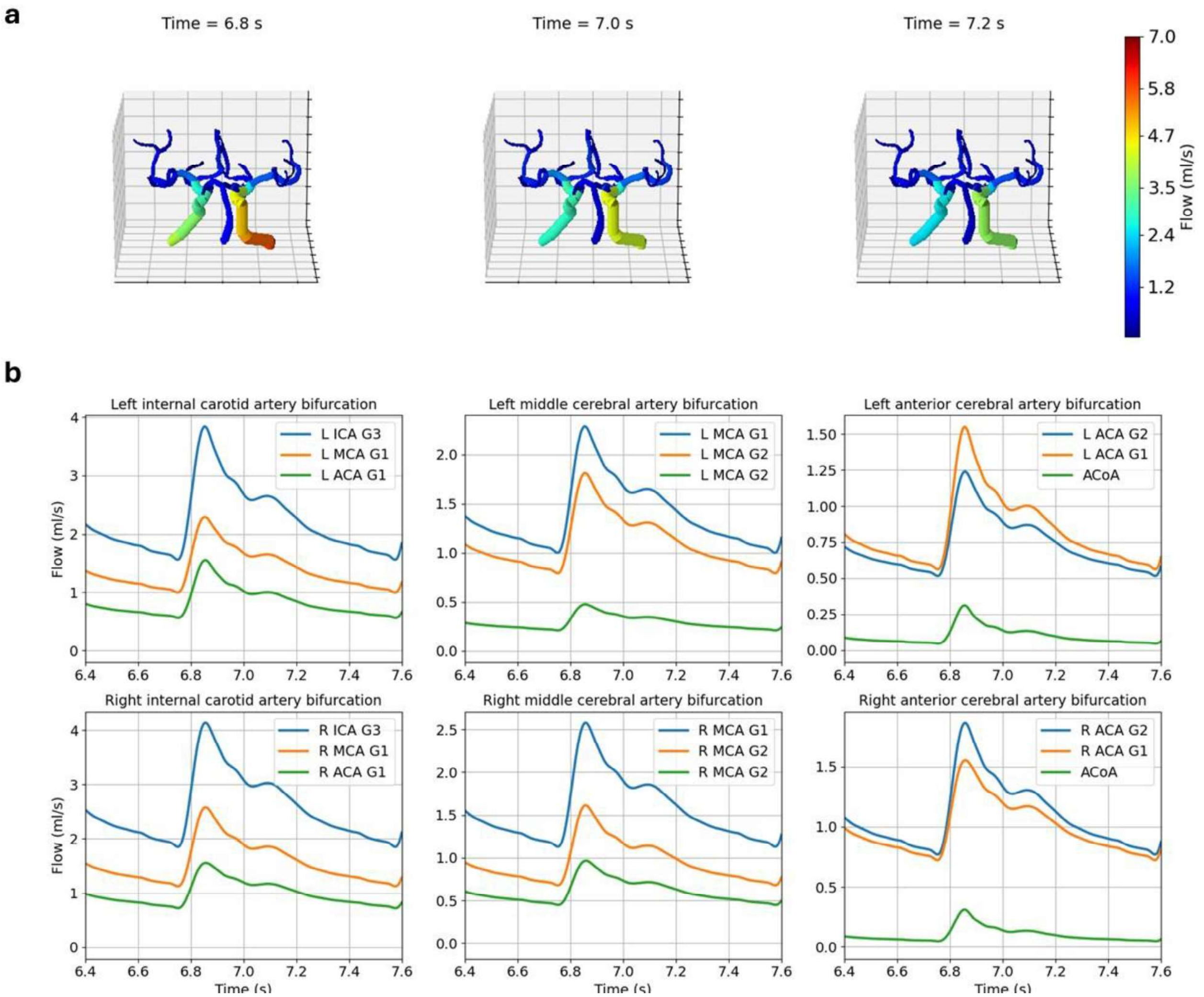

Figure 4 Blood flow distribution for Patient 5, Scan 2. Snapshots of flow distribution across the whole CoW at three different time points (a). Blood flow in time at different locations of the patient network (b).

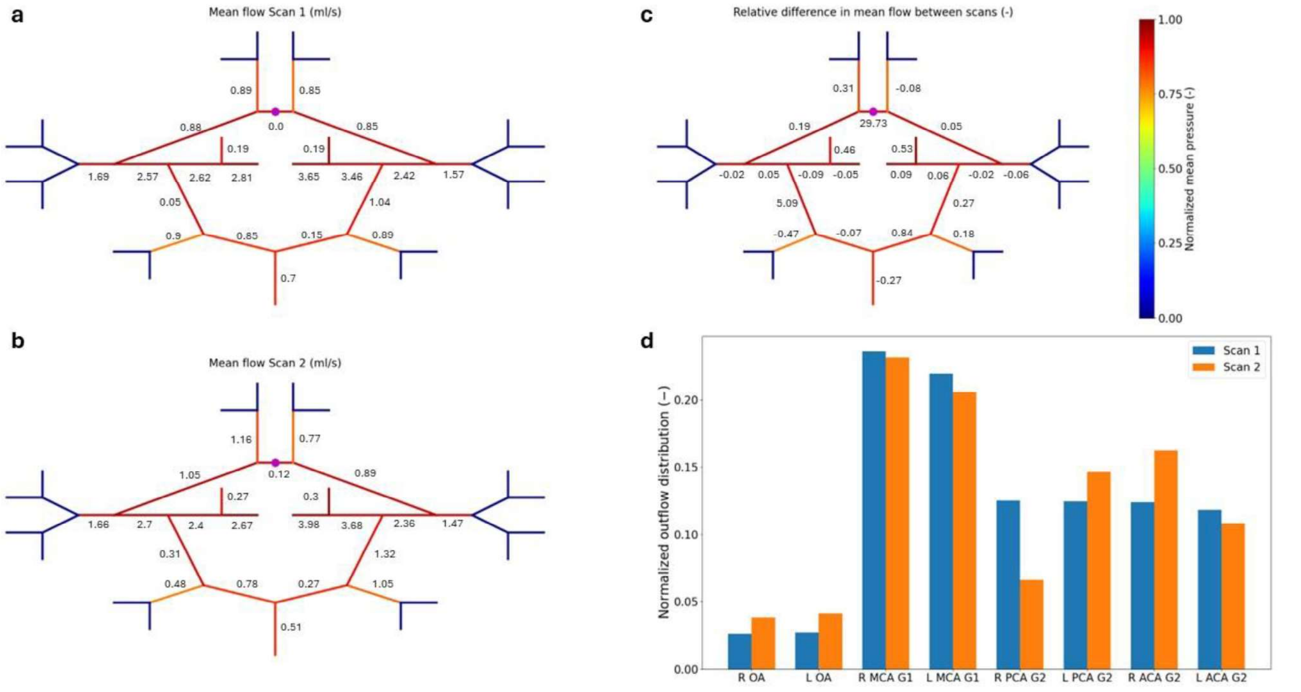

Figure 5 Blood flow distribution across CoW of Patient 5. Computed average blood flow in the vascular network derived from Scan 1 (a). Computed average blood flow in the vascular network derived from Scan 2 (b). Relative variation in average blood flow between scans (c). Normalized average blood flow distribution among outlets (d). The vessels are colored according to their mean pressure level. The aneurysm location is indicated with a purple full circle (stable). Normalized mean pressure is evaluated by considering all the vessels of the network as $\bar{P}_{norm} = (\bar{P} - min(\bar{P}))/(max(\bar{P}) - min(\bar{P}))$.

Relative variation of peak values across patient cohort

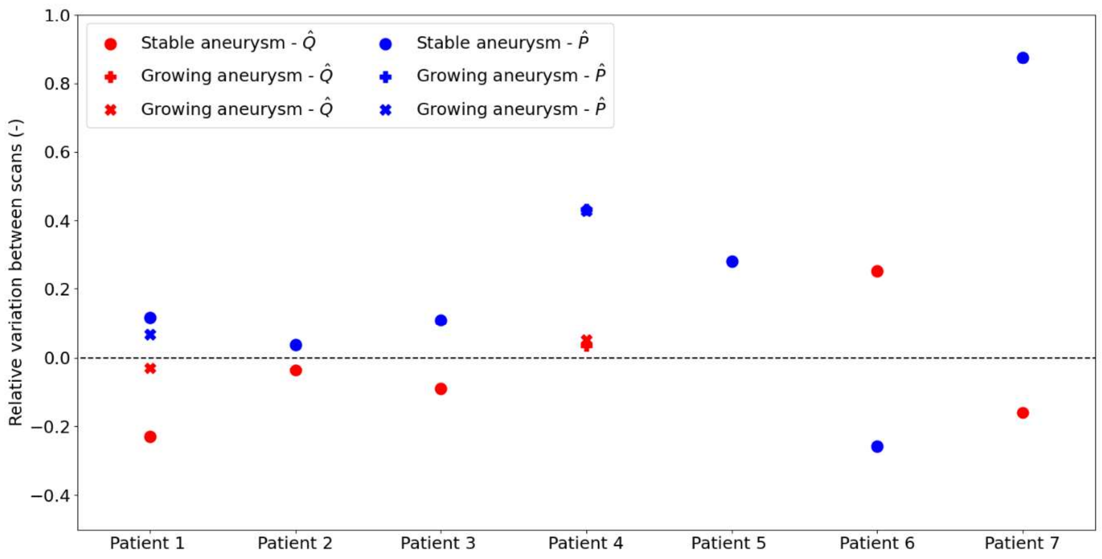

Figure 6 Relative variation of peak flow and pressure at the vessel with aneurysm across the patient cohort. The relative change in peak flow for Patient 5 is 2.02.
